# Analysis of input-output relationships of CPG elements and their contributions to rhythmic output

**DOI:** 10.1186/1471-2202-13-S1-P171

**Published:** 2012-07-16

**Authors:** Terrence Michael Wright, Brian Mulloney, Ronald L Calabrese

**Affiliations:** 1 Department of Biology, Emory University, Atlanta, GA 30322, USA; 2 Department of Neurobiology, Physiology and Behavior, University of California, Davis, Davis, CA, 95616, CA, USA

## 

**Figure 1:**
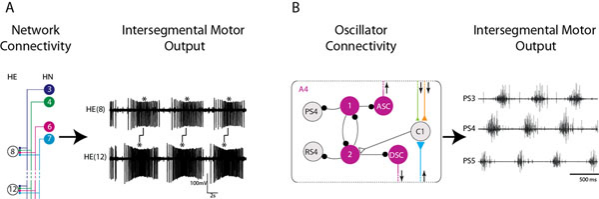
Circuit diagrams and rhythmic motor output from the leech heartbeat central pattern generator (A) and the crayfish swimmeret system (B). A, left: Heart (HE) motor neurons in segments 8 and 12 both receive synaptic input from premotor hear (HN) interneurons located in segments 3,4,6 and 7, which sculpt stereotyped motor output as shown in the simultaneous extracellular recordings of HE motor neurons in segments 8 and 12, which illustrate the peristaltic motor phase progression (right panel). B, left: circuit diagram for the independent oscillator in segment 4 (A4) driving PS4 motor activity observed in the extracellular recordings (right panel; Return stroke [RS(4)] activity not shown). Coordinating interneurons ASC and DSC in A4 encode information that is delivered to an interneuron (C1) in segments 3 (ASC) and 5 (DSC), resulting in the stereotyped rear-to-front activity pattern observed across segments 5,4 and 3 (right panel).

We use the dynamic clamp technique [1] to explore how synaptic input patterns affect motor output (see Figure 1A). We show that leech motor neuron intrinsic properties make a contribution to their output phasing. Then, we show that leech motor neurons receiving the same complement of synaptic inputs can still be organized into a coordinated motor pattern given that a gradient of synaptic strengths exists and that the premotor interneurons fire at different times [2]. In both of these cases, measuring motor neuron responses provides a direct assay for how premotor input patterns produce stereotyped motor output. We are currently extending this analysis to the crayfish swimmeret system, a system in which four segmental oscillators are interconnected by coordinating interneurons to produce a metachronal wave of swimmeret movements [3]. In this system, ascending (ASC) and descending (DSC) coordinating interneurons (see Figure 1B) encode salient features about the activity pattern of their home segment and are exported (via spikes) to other segmental oscillators. Interestingly, when the system is driven across periods by different concentrations of neuromodulators, the number of spikes of a given coordinating interneuron remains constant although their duty cycles change. We are currently building a single-compartment, conductance-based model of the ASC and DSC coordinating neurons, with the goal of understanding how these coordinating neurons encode information in their home modules and how this encoding can be modulated when the swimmeret system is driven at different periods.
